# Cortical thickness across the lifespan: Data from 17,075 healthy individuals aged 3–90 years

**DOI:** 10.1002/hbm.25364

**Published:** 2021-02-17

**Authors:** Sophia Frangou, Amirhossein Modabbernia, Steven C. R. Williams, Efstathios Papachristou, Gaelle E. Doucet, Ingrid Agartz, Moji Aghajani, Theophilus N. Akudjedu, Anton Albajes‐Eizagirre, Dag Alnæs, Kathryn I. Alpert, Micael Andersson, Nancy C. Andreasen, Ole A. Andreassen, Philip Asherson, Tobias Banaschewski, Nuria Bargallo, Sarah Baumeister, Ramona Baur‐Streubel, Alessandro Bertolino, Aurora Bonvino, Dorret I. Boomsma, Stefan Borgwardt, Josiane Bourque, Daniel Brandeis, Alan Breier, Henry Brodaty, Rachel M. Brouwer, Jan K. Buitelaar, Geraldo F. Busatto, Randy L. Buckner, Vincent Calhoun, Erick J. Canales‐Rodríguez, Dara M. Cannon, Xavier Caseras, Francisco X. Castellanos, Simon Cervenka, Tiffany M. Chaim‐Avancini, Christopher R. K. Ching, Victoria Chubar, Vincent P. Clark, Patricia Conrod, Annette Conzelmann, Benedicto Crespo‐Facorro, Fabrice Crivello, Eveline A. Crone, Anders M. Dale, Udo Dannlowski, Christopher Davey, Eco J. C. de Geus, Lieuwe de Haan, Greig I. de Zubicaray, Anouk den Braber, Erin W. Dickie, Annabella Di Giorgio, Nhat Trung Doan, Erlend S. Dørum, Stefan Ehrlich, Susanne Erk, Thomas Espeseth, Helena Fatouros‐Bergman, Simon E. Fisher, Jean‐Paul Fouche, Barbara Franke, Thomas Frodl, Paola Fuentes‐Claramonte, David C. Glahn, Ian H. Gotlib, Hans‐Jörgen Grabe, Oliver Grimm, Nynke A. Groenewold, Dominik Grotegerd, Oliver Gruber, Patricia Gruner, Rachel E. Gur, Ruben C. Gur, Tim Hahn, Ben J. Harrison, Catharine A. Hartman, Sean N. Hatton, Andreas Heinz, Dirk J. Heslenfeld, Derrek P. Hibar, Ian B. Hickie, Beng‐Choon Ho, Pieter J. Hoekstra, Sarah Hohmann, Avram J. Holmes, Martine Hoogman, Norbert Hosten, Fleur M. Howells, Hilleke E. Hulshoff Pol, Chaim Huyser, Neda Jahanshad, Anthony James, Terry L. Jernigan, Jiyang Jiang, Erik G. Jönsson, John A. Joska, Rene Kahn, Andrew Kalnin, Ryota Kanai, Marieke Klein, Tatyana P. Klyushnik, Laura Koenders, Sanne Koops, Bernd Krämer, Jonna Kuntsi, Jim Lagopoulos, Luisa Lázaro, Irina Lebedeva, Won Hee Lee, Klaus‐Peter Lesch, Christine Lochner, Marise W. J. Machielsen, Sophie Maingault, Nicholas G. Martin, Ignacio Martínez‐Zalacaín, David Mataix‐Cols, Bernard Mazoyer, Colm McDonald, Brenna C. McDonald, Andrew M. McIntosh, Katie L. McMahon, Genevieve McPhilemy, Susanne Meinert, José M. Menchón, Sarah E. Medland, Andreas Meyer‐Lindenberg, Jilly Naaijen, Pablo Najt, Tomohiro Nakao, Jan E. Nordvik, Lars Nyberg, Jaap Oosterlaan, Víctor Ortiz‐García de la Foz, Yannis Paloyelis, Paul Pauli, Giulio Pergola, Edith Pomarol‐Clotet, Maria J. Portella, Steven G. Potkin, Joaquim Radua, Andreas Reif, Daniel A. Rinker, Joshua L. Roffman, Pedro G. P. Rosa, Matthew D. Sacchet, Perminder S. Sachdev, Raymond Salvador, Pascual Sánchez‐Juan, Salvador Sarró, Theodore D. Satterthwaite, Andrew J. Saykin, Mauricio H. Serpa, Lianne Schmaal, Knut Schnell, Gunter Schumann, Kang Sim, Jordan W. Smoller, Iris Sommer, Carles Soriano‐Mas, Dan J. Stein, Lachlan T. Strike, Suzanne C. Swagerman, Christian K. Tamnes, Henk S. Temmingh, Sophia I. Thomopoulos, Alexander S. Tomyshev, Diana Tordesillas‐Gutiérrez, Julian N. Trollor, Jessica A. Turner, Anne Uhlmann, Odile A. van den Heuvel, Dennis van den Meer, Nic J. A. van der Wee, Neeltje E. M. van Haren, Dennis van 't Ent, Theo G. M. van Erp, Ilya M. Veer, Dick J. Veltman, Aristotle Voineskos, Henry Völzke, Henrik Walter, Esther Walton, Lei Wang, Yang Wang, Thomas H. Wassink, Bernd Weber, Wei Wen, John D. West, Lars T. Westlye, Heather Whalley, Lara M. Wierenga, Katharina Wittfeld, Daniel H. Wolf, Amanda Worker, Margaret J. Wright, Kun Yang, Yulyia Yoncheva, Marcus V. Zanetti, Georg C. Ziegler, Karolinska Schizophrenia Project (KaSP), Paul M. Thompson, Danai Dima

**Affiliations:** ^1^ Department of Psychiatry Icahn School of Medicine at Mount Sinai New York City New York USA; ^2^ Department of Psychiatry, Djavad Mowafaghian Centre for Brain Health University of British Columbia Vancouver Canada; ^3^ Department of Neuroimaging Institute of Psychiatry, Psychology and Neuroscience, King's College London London United Kingdom; ^4^ Psychology and Human Development Institute of Education, University College London London United Kingdom; ^5^ Institute for Human Neuroscience Boys Town National Research Hospital Omaha Nebraska USA; ^6^ Norwegian Centre for Mental Disorders Research (NORMENT) Institute of Clinical Medicine, University of Oslo Oslo Norway; ^7^ Department of Psychiatric Research Diakonhjemmet Hospital Oslo Norway; ^8^ Centre for Psychiatric Research, Department of Clinical Neuroscience Karolinska Institutet Solna Sweden; ^9^ Department of Psychiatry Amsterdam University Medical Centre, Vrije Universiteit Amsterdam Netherlands; ^10^ Section Forensic Family & Youth Care Institute of Education & Child Studies Leiden University Netherlands; ^11^ Institute of Medical Imaging and Visualisation, Department of Medical Science and Public Health, Faculty of Health and Social Sciences Bournemouth University Poole United Kingdom; ^12^ Clinical Neuroimaging Laboratory, Centre for Neuroimaging and Cognitive Genomics and NCBES Galway Neuroscience Centre National University of Ireland Galway Ireland; ^13^ FIDMAG Germanes Hospitalàries Barcelona Spain; ^14^ Mental Health Research Networking Center (CIBERSAM) Madrid Spain; ^15^ Division of Mental Health and Addiction Institute of Clinical Medicine, University of Oslo Oslo Norway; ^16^ Radiologics, Inc Saint Louis Missouri USA; ^17^ Department of Integrative Medical Biology Umeå University Umeå Sweden; ^18^ Department of Psychiatry, Carver College of Medicine The University of Iowa Iowa City Iowa USA; ^19^ Social, Genetic and Developmental Psychiatry Centre Institute of Psychiatry, Psychology and Neuroscience, King's College London London United Kingdom; ^20^ Department of Child and Adolescent Psychiatry and Psychotherapy, Central Institute of Mental Health Heidelberg University Heidelberg Germany; ^21^ Imaging Diagnostic Centre Hospital Clinic, Barcelona University Clinic Barcelona Spain; ^22^ August Pi i Sunyer Biomedical Research Institut (IDIBAPS) Barcelona Spain; ^23^ Department of Psychology, Biological Psychology, Clinical Psychology and Psychotherapy University of Würzburg Würzburg Germany; ^24^ Department of Basic Medical Science, Neuroscience and Sense Organs University of Bari Aldo Moro Bari Italy; ^25^ Department of Biological Psychology Vrije Universiteit Amsterdam Netherlands; ^26^ Department of Psychiatry & Psychotherapy University of Lübeck Lübeck Germany; ^27^ Department of Psychiatry University of Pennsylvania Philadelphia Pennsylvania USA; ^28^ Department of Radiology and Imaging Sciences Indiana University School of Medicine Indianapolis Indiana USA; ^29^ Centre for Healthy Brain Ageing, School of Psychiatry University of New South Wales Kensington New South Wales Australia; ^30^ Rudolf Magnus Institute of Neuroscience University Medical Center Utrecht Utrecht Netherlands; ^31^ Donders Center of Medical Neurosciences Radboud University Nijmegen Netherlands; ^32^ Donders Centre for Cognitive Neuroimaging Radboud University Nijmegen Netherlands; ^33^ Donders Institute for Brain, Cognition and Behaviour Radboud University Nijmegen Netherlands; ^34^ Laboratory of Psychiatric Neuroimaging, Departamento e Instituto de Psiquiatria, Hospital das Clinicas HCFMUSP, Faculdade de Medicina Universidade de São Paulo São Paulo Brazil; ^35^ Department of Psychology, Center for Brain Science Harvard University Cambridge Massachusetts USA; ^36^ Department of Psychiatry Massachusetts General Hospital Boston Massachusetts USA; ^37^ Tri‐Institutional Center for Translational Research in Neuroimaging and Data Science (TReNDS), Georgia State University, Georgia Institute of Technology Emory University, USA Neurology, Radiology, Psychiatry and Biomedical Engineering, Emory University Atlanta Georgia USA; ^38^ MRC Centre for Neuropsychiatric Genetics and Genomics Cardiff University Cardiff United Kingdom; ^39^ Department of Child and Adolescent Psychiatry New York University New York New York USA; ^40^ Stockholm Health Care Services Stockholm Sweden; ^41^ Imaging Genetics Center, Mark and Mary Stevens Neuroimaging and Informatics Institute, Keck School of Medicine University of Southern California Marina del Rey California USA; ^42^ Mind‐Body Research Group, Department of Neuroscience KU Leuven Leuven Belgium; ^43^ Department of Psychology University of New Mexico Albuquerque New Mexico USA; ^44^ Mind Research Network Albuquerque New Mexico USA; ^45^ Department of Psychiatry Université de Montréal Montreal Canada; ^46^ Department of Child and Adolescent Psychiatry, Psychosomatics and Psychotherapy University of Tübingen Tübingen Germany; ^47^ HU Virgen del Rocio, IBiS University of Sevilla Sevilla Spain; ^48^ Groupe d'Imagerie Neurofonctionnelle, Institut des Maladies Neurodégénératives, UMR5293 Université de Bordeaux Bordeaux France; ^49^ Erasmus School of Social and Behavioural Sciences Erasmus University Rotterdam Rotterdam Netherlands; ^50^ Faculteit der Sociale Wetenschappen, Instituut Psychologie Universiteit Leiden Leiden Netherlands; ^51^ Center for Multimodal Imaging and Genetics, Department of Neuroscience University of California‐San Diego San Diego California USA; ^52^ Department of Radiology University of California‐San Diego San Diego California USA; ^53^ Department of Psychiatry and Psychotherapy University of Münster Germany; ^54^ Department of Psychiatry University of Melbourne Melbourne Australia; ^55^ Academisch Medisch Centrum Universiteit van Amsterdam Amsterdam Netherlands; ^56^ Faculty of Health, Institute of Health and Biomedical Innovation Queensland University of Technology Queensland Australia; ^57^ Kimel Family Translational Imaging Genetics Laboratory, Campbell Family Mental Health Research Institute CAMH Campbell Canada; ^58^ Department of Psychiatry University of Toronto Toronto Canada; ^59^ Biological Psychiatry Lab Fondazione IRCCS Casa Sollievo della Sofferenza San Giovanni Rotondo (FG) Italy; ^60^ Department of Psychology University of Oslo Oslo Norway; ^61^ Sunnaas Rehabilitation Hospital HT Nesodden Norway; ^62^ Division of Psychological and Social Medicine and Developmental Neurosciences Technische Universität Dresden Dresden Germany; ^63^ Faculty of Medicine Universitätsklinikum Carl Gustav Carus an der TU Dresden Dresden Germany; ^64^ Division of Mind and Brain Research, Department of Psychiatry and Psychotherapy Charité‐Universitätsmedizin Berlin Berlin Germany; ^65^ Bjørknes College Oslo Norway; ^66^ Language and Genetics Department Max Planck Institute for Psycholinguistics Nijmegen Netherlands; ^67^ Department of Psychiatry and Mental Health University of Cape Town Cape Town South Africa; ^68^ Department of Human Genetics Radboud University Medical Center Nijmegen Netherlands; ^69^ Department of Psychiatry Radboud University Medical Center Nijmegen Netherlands; ^70^ Department of Psychiatry and Psychotherapy Otto von Guericke University Magdeburg Magdeburg Germany; ^71^ Department of Psychiatry Tommy Fuss Center for Neuropsychiatric Disease Research Boston Children's Hospital, Harvard Medical School Boston Massachusetts USA; ^72^ Department of Psychology Stanford University Stanford California USA; ^73^ Department of Psychiatry and Psychotherapy University Medicine Greifswald, University of Greifswald Greifswald Germany; ^74^ German Center for Neurodegenerative Diseases (DZNE) Site Rostock/Greifswald Greifswald Germany; ^75^ Department for Psychiatry, Psychosomatics and Psychotherapy Universitätsklinikum Frankfurt, Goethe Universitat Frankfurt Germany; ^76^ Neuroscience Institute University of Cape Town Cape Town South Africa; ^77^ Section for Experimental Psychopathology and Neuroimaging, Department of General Psychiatry Heidelberg University Heidelberg Germany; ^78^ Department of Psychiatry Yale University New Haven Connecticut USA; ^79^ Learning Based Recovery Center VA Connecticut Health System West Haven Connecticut USA; ^80^ Lifespan Brain Institute, Perelman School of Medicine University of Pennsylvania Philadelphia Pennsylvania USA; ^81^ Children's Hospital of Philadelphia University of Pennsylvania Philadelphia Pennsylvania USA; ^82^ Melbourne Neuropsychiatry Center University of Melbourne Melbourne Australia; ^83^ Interdisciplinary Center Psychopathology and Emotion regulation University Medical Center Groningen, University of Groningen Groningen Netherlands; ^84^ Brain and Mind Centre University of Sydney Sydney Australia; ^85^ Departments of Experimental and Clinical Psychology Vrije Universiteit Amsterdam Amsterdam Netherlands; ^86^ Personalized Healthcare, Genentech, Inc. South San Francisco California USA; ^87^ Department of Psychiatry University Medical Center Groningen, University of Groningen Groningen Netherlands; ^88^ Department of Psychology Yale University New Haven Connecticut USA; ^89^ Norbert Institute of Diagnostic Radiology and Neuroradiology University Medicine Greifswald, University of Greifswald Greifswald Germany; ^90^ De Bascule, Academic Centre for Children and Adolescent Psychiatry Amsterdam Netherlands; ^91^ Department of Psychiatry Oxford University Oxford United Kingdom; ^92^ Center for Human Development, Departments of Cognitive Science, Psychiatry, and Radiology University of California San Diego California USA; ^93^ Department of Radiology Ohio State University College of Medicine Columbus Ohio USA; ^94^ Department of Neuroinformatics Araya, Inc. Tokyo Japan; ^95^ Department of Psychiatry University of California San Diego San Diego California USA; ^96^ Mental Health Research Center Russian Academy of Medical Sciences Moscow Russia; ^97^ Sunshine Coast Mind and Neuroscience Thompson Institute, University of the Sunshine Coast Queensland Australia; ^98^ Department of Child and Adolescent Psychiatry and Psychology Hospital Clinic, University of Barcelona Barcelona Spain; ^99^ Department of Psychiatry, Psychosomatics and Psychotherapy Julius‐Maximilians Universität Würzburg Würzburg Germany; ^100^ SA MRC Unit on Risk and Resilience in Mental Disorders, Department of Psychiatry Stellenbosch University Stellenbosch South Africa; ^101^ Queensland Institute of Medical Research Berghofer Medical Research Institute Queensland Australia; ^102^ Department of Psychiatry Bellvitge University Hospital‐IDIBELL, University of Barcelona Barcelona Spain; ^103^ Division of Psychiatry University of Edinburgh Edinburgh United Kingdom; ^104^ School of Clinical Sciences, Institute of Health and Biomedical Innovation Queensland University of Technology Queensland Australia; ^105^ Department of Psychiatry and Psychotherapy Central Institute of Mental Health, Heidelberg University Heidelberg Germany; ^106^ Department of Clinical Medicine Kyushu University Fukuoka Japan; ^107^ CatoSenteret Rehabilitation Hospital Son Norway; ^108^ Department of Radiation Sciences Umeå Center for Functional Brain Imaging, Umeå University Umeå Sweden; ^109^ Department of Clinical Neuropsychology Amsterdam University Medical Centre, Vrije Universiteit Amsterdam Amsterdam Netherlands; ^110^ Department of Psychiatry University Hospital “Marques de Valdecilla”, Instituto de Investigación Valdecilla (IDIVAL) Santander Spain; ^111^ Centro de Investigación Biomédica en Red de Salud Mental (CIBERSAM) Instituto de Salud Carlos III Madrid Spain; ^112^ Centre of Mental Health University of Würzburg Würzburg Germany; ^113^ Department of Psychiatry Hospital de la Santa Creu i Sant Pau, Institut d'Investigació Biomèdica Sant Pau, Universitat Autònoma de Barcelona Barcelona Spain; ^114^ Department of Psychiatry University of California at Irvine Irvine California USA; ^115^ Department of Psychosis Studies Institute of Psychiatry, Psychology & Neuroscience, King's College London London United Kingdom; ^116^ Center for Depression, Anxiety, and Stress Research McLean Hospital, Harvard Medical School Boston Massachusetts USA; ^117^ Centro de Investigacion Biomedica en Red en Enfermedades Neurodegenerativas (CIBERNED) Valderrebollo Spain; ^118^ Orygen The National Centre of Excellence in Youth Mental Health Melbourne Australia; ^119^ Centre for Youth Mental Health The University of Melbourne Melbourne Australia; ^120^ Department of Psychiatry and Psychotherapy University Medical Center Göttingen Göttingen Germany; ^121^ Centre for Population Neuroscience and Precision Medicine Institute of Psychiatry, Psychology & Neuroscience, King's College London London United Kingdom; ^122^ Department of General Psychiatry Institute of Mental Health Singapore Singapore; ^123^ Center for Genomic Medicine Massachusetts General Hospital Boston Massachusetts USA; ^124^ Department of Biomedical Sciences of Cells and Systems, Rijksuniversiteit Groningen University Medical Center Groningen Groningen Netherlands; ^125^ Queensland Brain Institute University of Queensland Queensland Australia; ^126^ PROMENTA Research Center, Department of Psychology University of Oslo Oslo Norway; ^127^ Neuroimaging Unit, Technological Facilities Valdecilla Biomedical Research Institute IDIVAL Cantabria Spain; ^128^ College of Arts and Sciences Georgia State University Atlanta Georgia USA; ^129^ School of Mental Health and Neuroscience, Faculty of Health, Medicine and Life Sciences Maastricht University Maastricht Netherlands; ^130^ Department of Psychiatry Leiden University Medical Center Leiden Netherlands; ^131^ Leiden Institute for Brain and Cognition Leiden University Medical Center Leiden Netherlands; ^132^ Department of Child and Adolescent Psychiatry/Psychology Erasmus University Medical Center, Sophia Children's Hospital Rotterdam The Netherlands; ^133^ Center for the Neurobiology of Learning and Memory University of California Irvine Irvine California USA; ^134^ Institute of Community Medicine University Medicine, Greifswald, University of Greifswald Greifswald Germany; ^135^ German Centre for Cardiovascular Research (DZHK), partner site Greifswald Greifswald Germany; ^136^ German Center for Diabetes Research (DZD), partner site Greifswald Greifswald Germany; ^137^ Department of Psychology University of Bath Bath United Kingdom; ^138^ Department of Psychiatry and Behavioral Sciences, Feinberg School of Medicine Northwestern University Evanston Illinois USA; ^139^ Department of Radiology Medical College of Wisconsin Milwaukee Wisconsin USA; ^140^ Institute for Experimental Epileptology and Cognition Research University of Bonn Bonn Germany; ^141^ Developmental and Educational Psychology Unit, Institute of Psychology Leiden University Leiden Netherlands; ^142^ National High Magnetic Field Laboratory Florida State University Tallahassee Florida USA; ^143^ Department of Child and Adolescent Psychiatry, Child Study Center NYU Langone Health New York City New York USA; ^144^ Instituto de Ensino e Pesquisa Hospital Sírio‐Libanês São Paulo Brazil; ^145^ Division of Molecular Psychiatry, Center of Mental Health University of Würzburg Würzburg Germany; ^146^ Department of Psychology, School of Arts and Social Sciences City University of London London United Kingdom

**Keywords:** aging, cortical thickness, development, trajectories

## Abstract

Delineating the association of age and cortical thickness in healthy individuals is critical given the association of cortical thickness with cognition and behavior. Previous research has shown that robust estimates of the association between age and brain morphometry require large‐scale studies. In response, we used cross‐sectional data from 17,075 individuals aged 3–90 years from the Enhancing Neuroimaging Genetics through Meta‐Analysis (ENIGMA) Consortium to infer age‐related changes in cortical thickness. We used fractional polynomial (FP) regression to quantify the association between age and cortical thickness, and we computed normalized growth centiles using the parametric Lambda, Mu, and Sigma method. Interindividual variability was estimated using meta‐analysis and one‐way analysis of variance. For most regions, their highest cortical thickness value was observed in childhood. Age and cortical thickness showed a negative association; the slope was steeper up to the third decade of life and more gradual thereafter; notable exceptions to this general pattern were entorhinal, temporopolar, and anterior cingulate cortices. Interindividual variability was largest in temporal and frontal regions across the lifespan. Age and its FP combinations explained up to 59% variance in cortical thickness. These results may form the basis of further investigation on normative deviation in cortical thickness and its significance for behavioral and cognitive outcomes.

## Graphical abstract

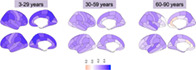

## 1 INTRODUCTION

In the last two decades, there has been a steady increase in the number of studies of age‐related changes in cerebral morphometry (Ducharme, et al., 2015; Good et al., 2001; Mutlu et al., 2013; Salat et al., 2004; Shaw et al., 2008; Storsve et al., 2014; Thambisetty et al., 2010; Wierenga, Langen, Oranje, & Durston, 2014) as a means to understand genetic and environmental influences on the human brain (Grasby, 2020; Modabbernia et al., 2020). Here we focus specifically on cortical thickness, as assessed using magnetic resonance imaging (MRI), as this measure has established associations with behavior and cognition in healthy populations (Goh et al., 2011; Schmitt et al., 2019; Shaw et al., 2006) and with disease mechanisms implicated in neuropsychiatric disorders (Boedhoe, et al., 2018; Hibar et al., 2018; Hoogman et al., 2019; Schmaal et al., 2017; Thompson et al., 2007; van Erp et al., 2018; van Rooij et al., 2018; Whelan et al., 2018).

Structural MRI is the most widely used neuroimaging method in research and clinical settings because of its excellent safety profile, ease of data acquisition and high patient acceptability. Thus, establishing the typical patterns of age‐related changes in cortical thickness as reference data could be a significant first step in the translational application of neuroimaging. The value of reference data is firmly established in medicine where deviations from an expected range are used to trigger further investigations or interventions. A classic example is the body mass index (BMI) which has been instrumental in informing about risk for relating to cardio‐metabolic outcomes (Aune et al., 2016).

There is significant uncertainty about the shape and interindividual variability of the association between age and cortical thickness. Prior studies have reported linear and nonlinear associations (e.g., Hedman, van Haren, Schnack, Kahn, & Hulshoff Pol, 2012; Mills et al., 2016) that may be influenced by sex (Paus, 2010; Raz, Ghisletta, Rodrigue, Kennedy, & Lindenberger, 2010; Wierenga et al., 2020). The present study harnessed the power of the Enhancing Neuroimaging Genetics through Meta‐Analysis (ENIGMA) Consortium, a multinational collaborative network of researchers organized into working groups, which conducts large‐scale analyses integrating data from over 250 institutions (Thompson et al., 2017; Thompson et al., 2020). Within ENIGMA, the focus of the Lifespan Working group is to delineate age‐associations in brain morphometric measures extracted from MRI images using standardized protocols and unified quality control procedures harmonized and validated across all participating sites. The ENIGMA Lifespan data set is the largest sample of healthy individuals available worldwide that offers the most comprehensive coverage of the human lifespan. This distinguishes the ENIGMA Lifespan data set from other imaging samples, such as the UK Biobank (http://www.ukbiobank.ac.uk) which includes individuals over 40 years of age. In the present study, we used MRI data from 17,075 healthy participants aged 3–90 years to infer age‐associated trajectories of cortical thickness. We also estimated regional interindividual variability in cortical thickness across the lifespan because it represents a major source of inter‐study variation (Raz et al., 2010; Wierenga et al., 2020). Based on prior literature, our initial hypotheses were that in most regions the relationship between age and thickness will follow an inverse U‐shape and will be influenced by sex.

## 2 MATERIALS AND METHODS

### 2.1 Study samples

**FIGURE 1:**
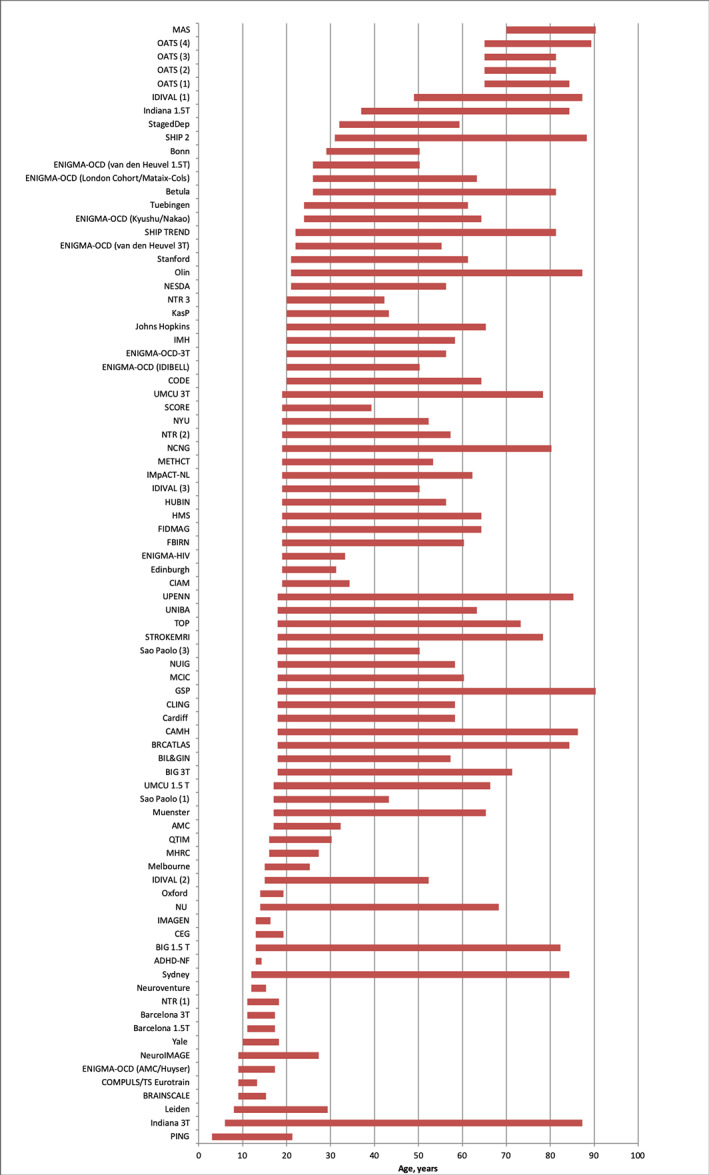
ENIGMA Lifespan samples. Abbreviations are explained in Table 1; further details of each sample are provided in the supplemental material

De‐identified demographic and cortical thickness data from 83 worldwide samples (Figure 1) were pooled to create the data set analyzed in this study. For samples from longitudinal studies, only baseline MRI scans were considered. The pooled sample comprised 17,075 participants (52% female) aged 3–90 years; only participants with complete data were included (Table 1). All participants had been screened to exclude psychiatric disorders, medical and neurological morbidity and cognitive impairment. Information on the screening protocols and eligibility criteria is provided in Table S1.

**TABLE 1:** Characteristics of the included samples

Sample	Age, mean, years	Age, *SD*, years	Age range		Sample *N*	Male *N*	Female *N*
ADHD NF	14	0.7	13	14	3	1	2
AMC	23	3.4	17	32	99	65	34
Barcelona 1.5 T	15	1.9	11	17	24	10	14
Barcelona 3 T	15	2.2	11	17	31	13	18
Betula	62	12.4	26	81	231	105	126
BIG 1.5 T	28	14.3	13	82	1,319	657	662
BIG 3 T	24	8.1	18	71	1,291	553	738
BIL&GIN	27	7.7	18	57	452	220	232
Bonn	39	6.5	29	50	175	175	0
BRAINSCALE	10	1.4	9	15	172	102	70
BRCATLAS	40	17.2	18	84	163	84	79
CAMH	44	19.3	18	86	141	72	69
Cardiff	26	7.8	18	58	265	78	187
CEG	16	1.8	13	19	31	31	0
CIAM	27	4.2	19	34	24	13	11
CLING	25	5.3	18	58	323	132	191
CODE	40	13.3	20	64	72	31	41
COMPULS/TS Eurotrain	11	1	9	13	42	29	13
Edinburgh	24	2.9	19	31	55	20	35
ENIGMA‐HIV	25	4.3	19	33	30	16	14
ENIGMA‐OCD (AMC/Huyser)	14	2.8	9	17	6	2	4
ENIGMA‐OCD (IDIBELL)	33	10.4	20	50	20	8	12
ENIGMA‐OCD (Kyushu/Nakao)	45	14.1	24	64	16	6	10
ENIGMA‐OCD (London Cohort/Mataix‐Cols)	38	11.6	26	63	10	2	8
ENIGMA‐OCD (van den Heuvel 1.5 T)	41	12.9	26	50	3	0	3
ENIGMA‐OCD (van den Heuvel 3 T)	36	10.9	22	55	8	4	4
ENIGMA‐OCD‐3 T‐CONTROLS	32	11	20	56	17	4	13
FBIRN	37	11.4	19	60	164	117	47
FIDMAG	38	10.1	19	64	123	54	69
GSP	27	16.5	18	90	2008	893	1,115
HMS	40	12.2	19	64	55	21	34
HUBIN	42	8.8	19	56	102	69	33
IDIVAL (1)	65	9.8	49	87	34	13	21
IDIVAL (3)	30	7.8	19	50	104	63	41
IDIVAL(2)	28	7.6	15	52	80	50	30
IMAGEN	14	0.4	13	16	1722	854	868
IMH	32	9.8	20	58	73	48	25
IMpACT‐NL	36	12.1	19	62	91	27	64
Indiana 1.5 T	62	11.7	37	84	49	9	40
Indiana 3 T	27	19.7	6	87	199	95	104
Johns Hopkins	44	12.5	20	65	85	42	43
KaSP	27	5.7	20	43	32	15	17
Leiden	17	4.8	8	29	572	279	293
MAS	79	4.7	70	90	385	176	209
MCIC	32	12.1	18	60	91	61	30
Melbourne	20	2.9	15	25	70	39	31
METHCT	27	6.5	19	53	39	29	10
MHRC	22	3.1	16	27	27	27	0
Muenster	35	12.1	17	65	744	323	421
NCNG	51	16.9	19	80	345	110	235
NESDA	40	9.7	21	56	65	23	42
NeuroIMAGE	17	3.4	9	27	252	115	137
Neuroventure	14	0.6	12	15	137	62	75
NTR (1)	15	1.4	11	18	37	14	23
NTR (2)	34	10.4	19	57	112	42	70
NTR (3)	30	5.9	20	42	29	11	18
NU	33	14.8	14	68	79	46	33
NUIG	36	11.5	18	58	92	53	39
NYU	31	8.7	19	52	51	31	20
OATS (1)	71	5.6	65	84	80	53	27
OATS (2)	69	5.1	65	81	13	7	6
OATS (3)	69	4	65	81	116	64	52
OATS (4)	70	4.7	65	89	90	63	27
Olin	36	13	21	87	582	231	351
Oxford	16	1.4	14	19	37	18	19
PING	12	4.8	3	21	431	223	208
QTIM	23	3.3	16	30	308	96	212
Sao Paolo	28	6.1	17	43	51	32	19
Sao Paolo‐2	31	7.6	18	50	58	30	28
SCORE	25	4.3	19	39	44	17	27
SHIP 2	55	12.3	31	88	306	172	134
SHIP TREND	50	13.7	22	81	628	355	273
StagedDep	48	8.1	32	59	23	7	16
Stanford	45	12.6	21	61	8	4	4
STROKEMRI	45	22.1	18	78	52	19	33
Sydney	39	22.1	12	84	157	65	92
TOP	35	9.9	18	73	303	159	144
Tuebingen	40	12.4	24	61	38	12	26
UMCU 1.5 T	33	12.5	17	66	278	158	120
UMCU 3 T	44	14	19	78	144	69	75
UNIBA	27	9.1	18	63	130	67	63
UPENN	37	13.1	18	85	115	42	73
Yale	14	2.7	10	18	12	5	7
Total	31	18.2	3	90	17,075	8,212	8,863

Abbreviations: ADHD‐NF, Attention Deficit Hyperactivity Disorder‐ Neurofeedback Study; AMC, Amsterdam Medisch Centrum; Basel, University of Basel; Barcelona, University of Barcelona; Betula, Swedish longitudinal study on aging, memory, and dementia; BIG, Brain Imaging Genetics; BIL&GIN, a multimodal multidimensional database for investigating hemispheric specialization; Bonn, University of Bonn; BrainSCALE, Brain Structure and Cognition: an Adolescence Longitudinal twin study; CAMH, Centre for Addiction and Mental Health; Cardiff, Cardiff University; CEG, Cognitive‐experimental and Genetic study of ADHD and Control Sibling Pairs; CIAM, Cortical Inhibition and Attentional Modulation study; CLiNG, Clinical Neuroscience Göttingen; CODE, formerly Cognitive Behavioral Analysis System of Psychotherapy (CBASP) study; Edinburgh, The University of Edinburgh; ENIGMA‐HIV, Enhancing NeuroImaging Genetics through Meta‐Analysis‐Human Immunodeficiency Virus Working Group; ENIGMA‐OCD, Enhancing NeuroImaging Genetics through Meta‐Analysis‐ Obsessive Compulsive Disorder Working Group; FBIRN, Function Biomedical Informatics Research Network; FIDMAG, Fundación para la Investigación y Docencia Maria Angustias Giménez; GSP, Brain Genomics Superstruct Project; HMS, Homburg Multidiagnosis Study; HUBIN, Human Brain Informatics; IDIVAL, Valdecilla Biomedical Research Institute; IMAGEN, the IMAGEN Consortium; IMH=Institute of Mental Health, Singapore; IMpACT, The International Multicentre persistent ADHD Genetics Collaboration; Indiana, Indiana University School of Medicine; Johns Hopkins, Johns Hopkins University; KaSP, The Karolinska Schizophrenia Project; Leiden, Leiden University; MAS, Memory and Aging Study; MCIC, MIND Clinical Imaging Consortium formed by the Mental Illness and Neuroscience Discovery (MIND) Institute now the Mind Research Network; Melbourne, University of Melbourne; Meth‐CT, study of methamphetamine users, University of Cape Town; MHRC, Mental Health Research Center; Muenster, Muenster University; NESDA, The Netherlands Study of Depression and Anxiety; NeuroIMAGE, Dutch part of the International Multicenter ADHD Genetics (IMAGE) study; Neuroventure: the imaging part of the Co‐Venture Trial funded by the Canadian Institutes of Health Research (CIHR); NCNG, Norwegian Cognitive NeuroGenetics sample; NTR, Netherlands Twin Register; NU, Northwestern University; NUIG, National University of Ireland Galway; NYU, New York University; OATS, Older Australian Twins Study; Olin, Olin Neuropsychiatric Research Center; Oxford, Oxford University; QTIM, Queensland Twin Imaging; Sao Paulo, University of Sao Paulo; SCORE, University of Basel Study; SHIP‐2 and SHIP TREND, Study of Health in Pomerania; Staged‐Dep, Stages of Depression Study; Stanford, Stanford University; StrokeMRI, Stroke Magnetic Resonance Imaging; Sydney, University of Sydney; TOP, Tematisk Område Psykoser (Thematically Organized Psychosis Research); TS‐EUROTRAIN, European‐Wide Investigation and Training Network on the Etiology and Pathophysiology of Gilles de la Tourette Syndrome; Tuebingen, University of Tuebingen; UMCU, Universitair Medisch Centrum Utrecht; UNIBA, University of Bari Aldo Moro; UPENN, University of Pennsylvania; Yale, Yale University.

### 2.2 Image acquisition and processing

Prior to pooling the data used in this study, researchers at each participating institution (a) used the ENIGMA MRI analysis protocols, which are based on FreeSurfer (http://surfer.nmr.mgh.harvard.edu), to compute the cortical thickness of 68 regions from high‐resolution T1‐weighted MRI brain scans collected at their site; (b) inspected all images by overlaying the cortical parcellations on the participants' anatomical scans and excluded improperly segmented scans; (c) identified outliers using five median absolute deviations (MAD) of the median value (additional details in the supplement). Information on scanner vendor, magnetic field strength, FreeSurfer version and acquisition parameters for each sample as provided by the participating institutions is detailed in Table S1.

### 2.3 Analysis of age‐related changes in cortical thickness

We modeled the effect of age on regional cortical thickness using higher order fractional polynomial (FP) regression analyses (Royston & Altman, 1994; Sauerbrei, Meier‐Hirmer, Benner, & Royston, 2006) implemented in STATA software version 14.0 (Stata Corp., College Station, TX). FP regression is one of the most flexible methods to study the effect of continuous variables on a response variable (Royston & Altman, 1994; Sauerbrei et al., 2006). FP allows for testing a broad family of shapes and multiple turning points while simultaneously providing a good fit at the extremes of the covariates (Royston & Altman, 1994). Prior to FP regression analysis, cortical thickness values were harmonized between sites using the ComBat method in R (Fortin et al., 2018). ComBat uses an empirical Bayes method to adjust for inter‐scanner variability in the data while preserving biological variability. As the effect of scanner was adjusted using ComBat, we only included sex as a covariate in the regression models. Additionally, standard errors were adjusted for the effect of scanner in the FP regression. We centered the data from each brain region so that the intercept of an FP was zero for all covariates. We used a predefined set of power terms (−2, −1, −0.5, 0.5, 1, 2, 3) and the natural logarithm function, and up to four power combinations to identify the best fitting model. FP for age was written as age^(p1, p2, …p6)′^β where p in *age* ^(*p*1, *p*2, …*p*6)^ refers to regular powers except *age* ^(0)^ which refers to ln(age). Powers can be repeated in FP; each time a power s repeated, it is multiplied by another ln(age). As an example:

$$\;{\mathrm{age}}^{\left(0,1,1\right)\prime }\beta =\beta_{0}+\beta_{1}{\mathrm{age}}^{0}+\beta_{2}{\mathrm{age}}^{1}+\beta_{3}{\mathrm{age}}^{1}\ln\left(\mathrm{age}\right)=\beta_{0}+\beta_{1}\ln\left(\mathrm{age}\right)+\beta_{2}\mathrm{age}+\beta_{3}\mathrm{age}\;\ln\left(\mathrm{age}\right)$$

494 models were trained for each region. Model comparison was performed using a partial *F*‐test and the lowest degree model with the smallest *p‐value* was selected as the optimal model. Following permutation, critical alpha value was set at .01 to decrease the probability of over‐fitting. The age at maximum cortical thickness for each cortical region was the maximum fitted value of the corresponding optimal FP model.

Further, we divided the data set into three age‐groups corresponding to early (3–29 years), middle (30–59 years) and late life (60–90 years). Within each age‐group, we calculated Pearson's correlation coefficient between age and regional cortical thickness. Finally, we used the *cocor* package in R to obtain P‐values for the differences in correlation coefficients between males and females in each age‐group.

### 2.4 Interindividual variation in cortical thickness

The residuals of the FP regression models for each cortical region were normally distributed. Using one‐way analysis of variance we extracted the residual variance around the optimal fitted FP regression model so as to identify age‐group differences in interindividual variation for each cortical region. Separately for each age‐group (t), we calculated the mean age‐related variance of each cortical region using $\left(\frac{\sum \sqrt{e_{i}^{2}}}{n_{t}}\right)\;$ where *e* ^*2*^ denotes the squared residual variance of that region around the best fitting FP regression line for each individual (i) of that age‐group, and *n* the number of observations in that age‐group. Because the square root of the squared residuals was positively skewed, we applied a natural logarithm transformation to the calculated variance. To account for multiple comparisons (68 regions assessed in three age‐groups), a Bonferroni adjusted *p*‐value of 0.0007 as chosen as a cut‐off for a significant *F‐*test. To confirm that the scanner effect did not drive the interindividual variability analyses, we also conducted a meta‐analysis of the *SD* of the regional cortical thickness in each age‐group, following previously validated methodology (Senior, et al., 2016). To test whether interindividual variability is a function of surface area (and possibly measurement error by FreeSurfer) we plotted the *SD* values of each region against their corresponding average surface area.

### 2.5 Centile values of cortical thickness

We calculated the centiles (0.4, 1, 2.5, 5, 10, 25, 50, 75, 90, 95, 97.5, 99, 99.6) for each regional cortical thickness measure by sex and hemisphere as normalized growth centiles using parametric Lambda (λ), Mu (μ), Sigma (σ) (LMS) method (Cole and Green, 1992) in the Generalized Additive Models for Location, Scale and Shape (GAMLSS) package in R (http://cran.r-project.org/web/packages/gamlss/index.html) (Rigby & Stasinopoulos, 2005; Stasinopoulos & Rigby, 2007). LMS is considered a powerful method for estimating centile curves based on the distribution of a response variable at each covariate value (in this case age). GAMLSS uses a penalized maximum likelihood function to estimate parameters of smoothness (effective degrees of freedom) which are then used to estimate the λ, μ, and σ parameters. The goodness of fit for these parameters in the GAMLSS algorithm is established by minimizing the Generalized Akaike Information Criterion (GAIC) index.

## 3 RESULTS

### 3.1 Association of age with cortical thickness

**FIGURE 2:**
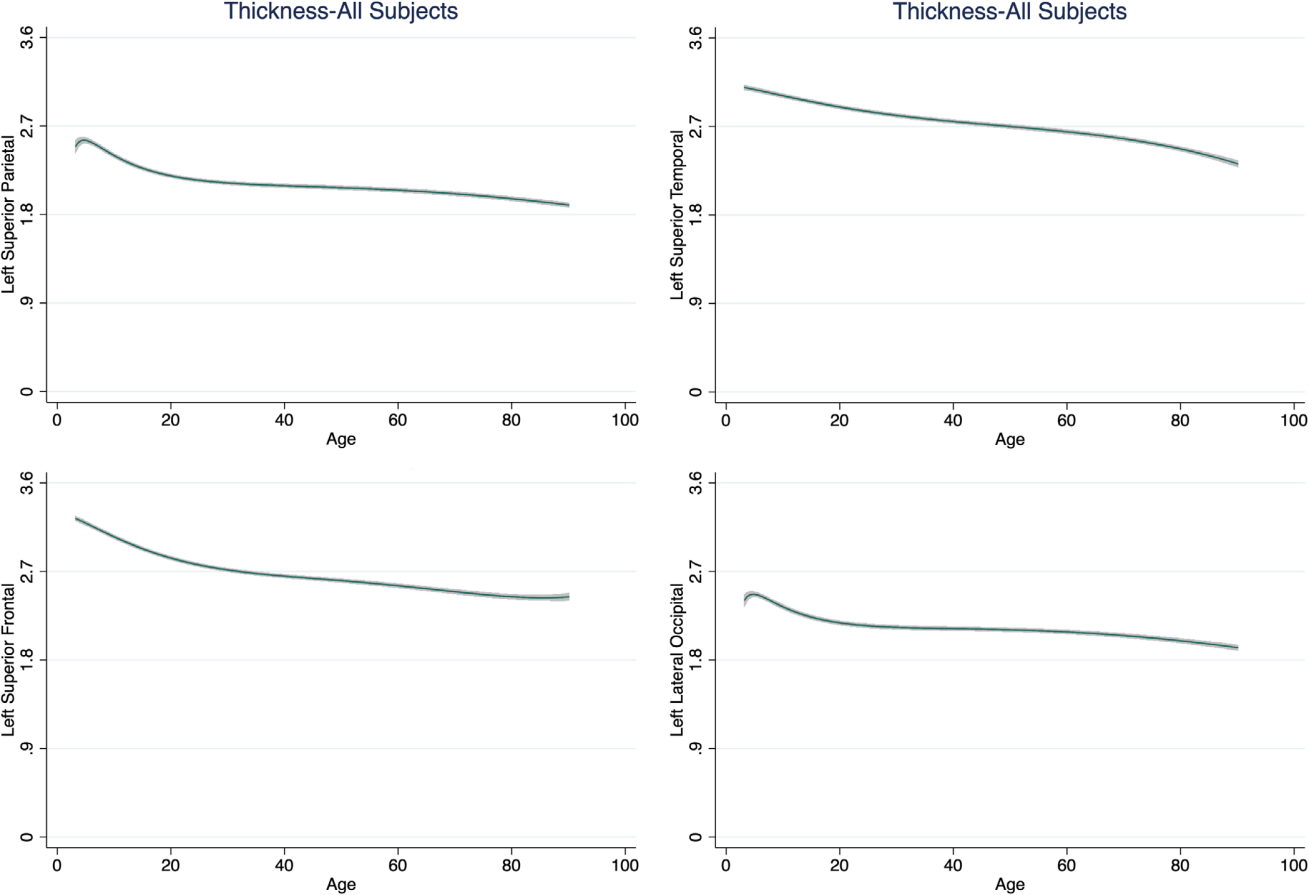
Illustrative Fractional Polynomial Plots for the association of age and cortical thickness. We present exemplars from each lobe as derived from fractional polynomial analyses of the entire data set. Details regarding the association of age and thickness for all cortical regions (for the entire data set and separately for males and females) are given in the supplementary material

Figure 2 shows the shape of the association of age with cortical thickness in each lobe, while the corresponding information on all cortical regions is provided in File S1. For most regions, the highest value for cortical thickness was observed in childhood; age and cortical thickness showed a negative linear correlation, with the slope being steep until the third decade of life (Table S2). By contrast, the entorhinal and temporopolar cortices showed an inverse U‐shaped relation with age bilaterally while in the anterior cingulate cortex (ACC) showed an attenuated U‐shape. In general, age and its FP combinations explained up to 59% of the variance in mean cortical thickness (Table S2). Age explained the smallest proportion of the variance for entorhinal (1–2%) and temporopolar (2–3%) cortices but the largest proportion of variance for the superior frontal and precuneus gyri (50–52%).

We observed significant sex differences in the slopes of age‐related mean cortical thickness reduction in the middle‐life group (30–59 years) which were steeper for males (*r* = −.39 to −.38) than for females (*r* = −.27). In the early‐life group (3–29 years), the age‐related slopes for mean cortical thickness were not different between males (*r* = −.59) and females (*r* = −.56). Similarly, in the late‐life group (61–90 years) there were no meaningful sex differences (male: *r*‐range = −.30 to −.29; female: *r*‐range= = − .33 to −.31).

**FIGURE 3:**
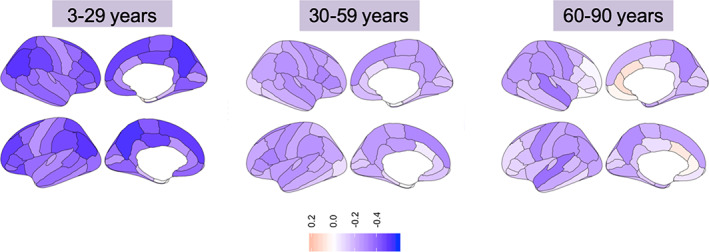
Correlation between age and cortical thickness across age‐groups. Left panel: early life age‐group (3–29 years); Middle panel: middle life age‐group (30–59 years); Right panel: late life age‐group (60–90 years). Blue hues = negative correlations; Red hues = positive correlations

Further, sex differences were also noted at the regional level in the early‐ and middle‐life groups. In the early‐life group, the slope of the association between age and cortical thickness was steeper in males than in females in the bilateral cuneus, lateral occipital, lingual, superior parietal, postcentral, and paracentral, precuneus, and pericalcarine gyri (all *p* < .0007). In middle‐life age‐group, the slope was steeper in males than in females in the bilateral *pars orbitalis* and *pars triangularis* as well as left isthmus of the cingulate, *pars opercularis*, precuneus, rostral middle frontal, and supramarginal, and right fusiform, inferior temporal, inferior parietal, lateral occipital, lateral orbitofrontal, rostral anterior cingulate, superior frontal, supramarginal regions, and the insula (all *p* < .0002) (Figures 3 and S1, Table S3).

### 3.2 Interindividual variation in cortical thickness

**FIGURE 4:**
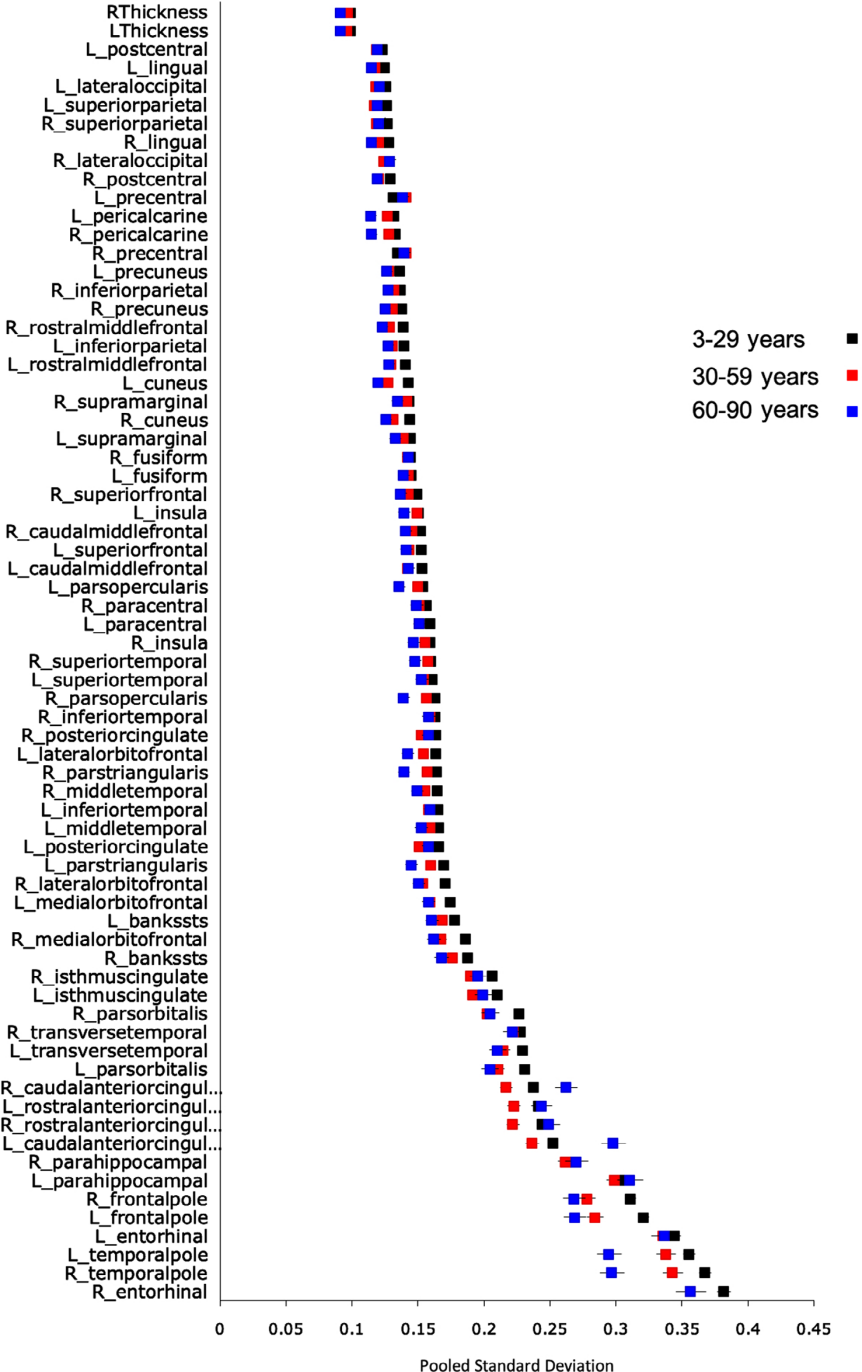
Interindividual variability in cortical thickness across the lifespan. The plot presents the pooled *SD* in regional cortical thickness values om the early, middle and late life age‐groups

Across age‐groups (early, middle, and late life), interindividual variability in regional cortical thickness, as measured by pooled *SD*, was between 0.1 and 0.2 mm. Details are provided in Table S4, Figures 4 and S2. High interindividual variation was mainly confined bilaterally in the entorhinal, parahippocampal, transverse temporal, temporopolar, frontopolar, anterior cingulate and isthmus, and *pars orbitalis regions*. We confirmed the replicability of these findings in each age‐group by conducting meta‐analysis following the procedures set‐out by Senior et al. (2016).

Finally, we observed a nonlinear association between regional cortical surface area and interindividual variability with variability being typically higher in regions with smaller surface areas (Figure S3).

### 3.3 Centile curves of cortical thickness

**FIGURE 5:**
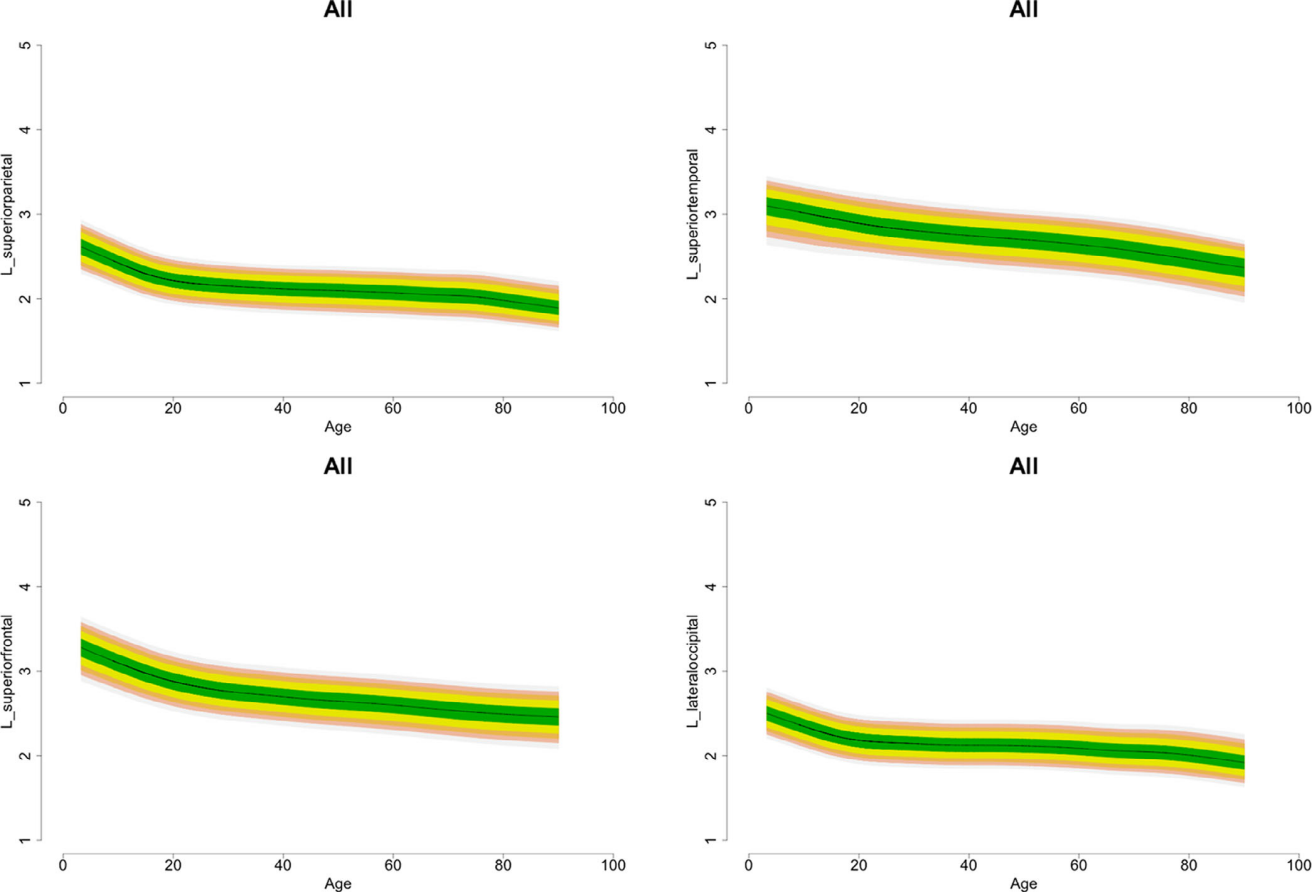
Illustrative normative centile curves of cortical thickness. We present exemplar sets of centile curves for each lobe as derived from LMS of the entire data set. Normative centile curves for all cortical regions (for the entire data set and separately for males and females) are given in the supplementary material

Representative centiles curves for each lobe are presented in Figure 5. Centile values for the thickness of each cortical region, stratified by sex and hemisphere, are provided in Tables S5 to S7 and File S2.

## 4 DISCUSSION

In the present study, we provide the most comprehensive characterization of the association between age and regional cortical thickness across the human lifespan based on multiple analytic methods (i.e., FP analysis, meta‐analysis and centile calculations) and the largest data set of cortical thickness measures available from healthy individuals aged 3 to 90 years. In addition to sample size, the study benefited from the standardized and validated protocols for data extraction and quality control that are shared by all ENIGMA sites and have supported all published ENIGMA structural MRI studies (Thompson et al., 2020).

Most regional cortical thickness measures reached their maximum value between 3 and 10 years of age, showed a steep decrease during the second and third decades of life and an attenuated or plateaued slope thereafter. This pattern was independent of the hemisphere and sex. A recent review (Walhovd, Fjell, Giedd, Dale, & Brown, 2017) has highlighted contradictions between studies that report an increase in cortical thickness during early childhood and studies that report a decrease in cortical thickness during the same period. The results from the current study help reconcile previous findings as they show that the median age at maximum thickness for most cortical regions is in the lower bound of the age‐range examined here. However, these findings must be considered in the context to the fewer data points available for those below the age of 10 years.

The general pattern of greater cortical thinning with advancing age was similar in both sexes. When participants were divided in early‐, middle‐ and late‐life groups, sex differences in the slope between age and cortical thickness was noted primarily for the mid‐life group. In this age‐group, which included individuals aged 30–59 years, the slope was steeper in males than in females. This sex‐difference has not been reported in other studies (Fjell et al., 2015; Raz et al., 2005; Raz et al., 2010; Storsve et al., 2014) which generally had smaller samples (<2000), shorter observation periods or examined age‐related trajectories of cortical thickness after the effect of sex was regressed‐out (e.g., Fjell et al., 2009). Although the sex‐differences reported here may be incidental, they resonate with findings of generally higher cognitive reserve in women as they enter later‐life (Mauvais‐Jarvis et al., 2020).

In the entorhinal and temporopolar cortex there were minimal age‐related changes until the seventh to eighth decades of life; thereafter both regions showed age‐related decrease in cortical thickness. Although the FreeSurfer estimation of cortical thickness in these regions is often considered suboptimal (compared with the rest of the brain), we note that our findings are consistent with a prior multicenter study of 1,660 healthy individuals (Hasan et al., 2016). Further, the current study supports results from the National Institutes of Health MRI study of 384 individuals that found no significant change in the bilateral entorhinal and medial temporopolar cortex between the ages of 4–22 years (Ducharme et al., 2016). A further study of 207 healthy adults aged 23–87 years also showed no significant cortical thinning in the entorhinal cortex until the sixth decade of life (Storsve et al., 2014). These observations suggest that the cortex of the entorhinal and temporopolar regions is largely preserved across the lifespan in healthy individuals. Both these regions contribute to episodic memory while the temporopolar region is also involved in semantic memory (Rolls, 2018). Degenerative changes of the temporopolar cortex have been reliably associated with semantic dementia, which is characterized by loss of conceptual knowledge about real‐world items (Hodges & Patterson, 2007). The integrity and resting metabolic rate of the temporopolar cortex decrease with age (Allen, Bruss, Brown, & Damasio, 2005; Eberling et al., 1995; Fjell et al., 2009), and lower perfusion rates in this region correlate with cognitive impairment in patients with Alzheimer's disease (AD) (Alegret et al., 2010). Entorhinal cortical thickness is a reliable marker of episodic memory performance (Schultz, Sommer, & Peters, 2012) and entorhinal cortex volume and metabolism are reduced in patients with AD and mild cognitive impairment (Dickerson et al., 2009; Zhou, Zhang, Zhao, Qian, & Dong, 2016). We therefore infer that “accelerated” entorhinal and temporopolar cortical thinning may be a marker of age‐related cognitive decline; as they grow older, individuals at risk of cognitive decline may show a gradual leftward shift in the distribution of the cortical thickness of these regions which coincides with the exponential age‐related increase in the incidence of AD in the later decades of life (Reitz & Mayeux, 2014).

The thickness of the ACC showed an attenuated U‐shaped association with age. This observation replicates an earlier finding in 178 healthy individuals aged 7–87 years (Sowell, et al., 2007). The U‐shaped age trajectory of ACC thickness might explain divergent findings in previous studies that have reported age‐related increases (Abe et al., 2008; Salat et al., 2004), age‐related reductions or no change (Brickman, Habeck, Zarahn, Flynn, & Stern, 2007; Ducharme et al., 2016; Good et al., 2001; Vaidya, Paradiso, Boles Ponto, McCormick, & Robinson, 2007).

A consistently higher degree of interindividual variation was observed in the most rostral frontal regions (frontopolar cortex and *pars orbitalis*), in the ACC and in several temporal regions (entorhinal, parahippocampal, temporopolar, and transverse temporal cortex). To some degree, greater variability in several of these regions may reflect measurement challenges associated with their small size (Figure S3). Nevertheless, the pattern observed suggests that greater interindividual variability may be a feature of proisocortical and periallocortical regions (in the cingulate and temporal cortices) that are anatomically connected to prefrontal isocortical regions, and particularly the frontopolar cortex. This prefrontal isocortical region is considered evolutionarily important based on its connectivity and function in humans and nonhuman primates (Ongür, Ferry, & Price, 2003; Semendeferi et al., 2011). The frontopolar region has several microstructural characteristics, such as a higher number and greater width of minicolumns and greater interneuron space, which are conducive to facilitating neuronal connectivity (Semendeferi et al., 2011). According to the popular “gateway” hypothesis, the lateral frontopolar cortex implements processing of external information (“stimulus‐oriented” processing) while the medial frontopolar cortex attends to self‐generated or maintained representations (“stimulus‐independent” processing) (Burgess, Dumontheil, & Gilbert, 2007). Stimulus‐oriented processing in the frontopolar cortex is focused on multitasking and goal‐directed planning while stimulus‐independent processing involves mainly metalizing and social cognition (Gilbert, Gonen‐Yaacovi, Benoit, Volle, & Burgess, 2010). The other regions (entorhinal, parahippocampal, cingulate, and temporopolar) with high interindividual variation in cortical thickness are periallocortical and proisocortical regions that are functionally connected to the medial frontopolar cortex (Gilbert et al., 2010; Moayedi, Salomons, Dunlop, Downar, & Davis, 2015). Notably, the periallocortex and proisocortex are considered transitional zones between the phylogenetically older allocortex and the more evolved isocortex. Specifically, the entorhinal cortex is perialiocortical (Insausti, Muñoz‐López, Insausti, & Artacho‐Pérula, 2017), the cingulate and parahippocampal cortices are proisocortical and the cortex of the temporopolar region is mixed (Blaizot et al., 2010; Petrides, Tomaiuolo, Yeterian, & Pandya, 2012). Considered together, these regions are core nodes of the default mode network (DMN; Raichle et al., 2001). At present, it is unclear whether this higher interindividual variation in the cortical thickness of the DMN nodes is associated with functional variation, but this is an important question for future studies.

The results presented here are based on the largest available brain MRI data set worldwide covering the human lifespan. However, none of the pooled samples in the current study was longitudinal. We fully appreciate that longitudinal studies are considered preferable to cross‐sectional designs when aiming to define age‐related brain morphometric trajectories. However, a longitudinal study of this size over nine decades of life is not feasible. In addition to problems with participant recruitment and retention, such a lengthy study would have involved changes in scanner types, magnetic field strengths, and acquisition protocols in line with necessary upgrades and technological advances. Nevertheless, it is possible to test the alignment between the results presented here and data from longitudinal cohorts, many of which are also available through the ENIGMA consortium. We consider this an important direction for follow‐up studies. We took several steps to mitigate against site effects. First, we ensured that we used age‐overlapping data sets throughout. Second, standardized analyses and quality control protocols were used to extract cortical thickness measures at all participating institutions. Third, we estimated and controlled for the contribution of site and scanner using ComBat prior to conducting our analysis. The validity of the findings reported here is reinforced by their alignment with the results from short‐term longitudinal studies of cortical thickness (Shaw et al., 2008; Storsve et al., 2014; Tamnes et al., 2010; Thambisetty et al., 2010; Wierenga et al., 2014). The generalizability of our findings for the older age‐group is qualified by our selection of individuals who appear to be aging successfully in terms of cognitive function and absence of significant medical morbidity. Nevertheless, despite the efforts to include only healthy older individuals, the observed pattern of brain aging may still be influenced by subclinical mental or medical conditions. For example, vascular risk factors (e.g., hypertension) are prevalent in older individuals and have been associated with decline in the age‐sensitive regions identified here (Raz et al., 2005). Thus, we cannot conclusively exclude the possibility that such factors may have contributed to our results. In addition, a wide range of factors have been associated with cortical morphology throughout the lifespan. Key among them are genetic factors (Grasby, 2020; Teeuw et al., 2019) and indices of socioeconomic status (Chan et al., 2018; Modabbernia et al., 2020; Ziegler et al., 2020) and possibly race (Zahodne et al., 2015). These factors were not modeled here as the relevant information was not collected in a systematic and harmonized fashion across contributing cohorts. It is therefore unclear to what extent they might have influenced the general pattern of age‐related associations with cortical thickness reported in the current study; qualifying their possible effects is a priority for future investigations. Cellular studies show that the number of neurons, the extent of dendritic arborization, and amount of glial support explain most of the variability in cortical thickness (la Fougère et al., 2011; Pelvig, Pakkenberg, Stark, & Pakkenberg, 2008; Terry, DeTeresa, & Hansen, 1987). MRI lacks the resolution to assess microstructural tissue properties but provides an estimate of cortical thickness based on the MR signal. Nevertheless, there is remarkable similarity between MRI‐derived thickness maps and postmortem data (Fischl & Dale, 2000). Finally, we present the centile curves to stimulate further research in developing normative reference values for neuroimaging phenotypes which should include investigation of measurement errors and reproducibility. In this context, the centile curves should not be used clinically or to make inferences about single individuals.

The findings of the current study suggest several avenues of further research. MRI‐derived measures of cortical thickness do not provide information on the mechanisms that underlie the observed age‐related associations. However, the results provided here could be used to study further factors that may lead to deviations in cortical thickness way from the expected age‐appropriate range. Additionally, the results of the current study provide a new avenue for investigating the functional correlates, either cognitive or behavioral, of age‐related changes and interindividual variation in regional cortical thickness.

In summary, using existing cross‐sectional data from 17,075 individuals we performed a large‐scale analysis to investigate the age‐related changes in cortical thickness. The size and age‐coverage of the analysis sample has the potential to inform about developmental and aging changes in cortical morphology and provide a foundation the study of factors that may lead to deviations from normative patterns.

## CONFLICT OF INTERESTS

Hans Jörgen Grabe: Travel grants and speaker honoraria from Fresenius Medical Care, Neuraxpharm, Servier and Janssen Cilag; reseach funding from Fresenius Medical Care. Ole A Andreasen: Consultant to HealthLytix, speaker honorarium from Lundbeck. Anders M Dale: Founder and member of the Scientific Advisory Board CorTechs Labs, Inc where he holds equity; member of the Scientific Advisory of Human Longevity Inc; research grants with General Electric Healthcare.

## Supporting information


**Figure S1** Histogram of age‐distribution across all samples
**Figure S2.** Correlation between age and cortical thickness across age‐groups and stratified by sex
**Figure S3.** Meta‐analysis of pooled standard deviation stratified by sex
**Figure S4.** Pooled Standard deviation of cortical regions as a function of surface area
**Table S1.** Screening Process and Eligibility Criteria, Scanner, Image Acquisition Parameters and Image Segmentation Software
**Table S2.** Variance Explained by Age in fractional polynomial model
**Table S3**: Pearson's Correlation Coefficient between Age and Cortical Thickness
**Table S4.** Interindividual variations in cortical thickness
**Table S5.** Centile Values for Cortical Thickness
**Table S6.** Centile Values for Cortical Thickness in Males
**Table S7.** Centile Values for Cortical Thickness in Females

**Appendix S1** Supporting Information.

**Appendix S2** Supporting Information.

## Data Availability

The ENIGMA Lifespan Working Group welcomes expression of interest from researchers in the field who wish to use the ENIGMA samples. Data sharing is possible subsequent to consent for the principal investigators of the contributing datasets. Requests should be directed to the corresponding authors.

## APPENDIX A.

Göran Engberg, Department of Physiology and Pharmacology, Karolinska Institute, Sweden.

Sophie Erhardt, Department of Physiology and Pharmacology, Karolinska Institute, Sweden.

Lilly Schwieler, Department of Physiology and Pharmacology, Karolinska Institute, Sweden.

Funda Orhan, Department of Physiology and Pharmacology, Karolinska Institute, Sweden.

Anna Malmqvist, Department of Physiology and Pharmacology, Karolinska Institute, Sweden. Mikael Hedberg, Department of Physiology and Pharmacology, Karolinska Institute, Sweden.

Lars Farde, Department of Clinical Neuroscience, Center for Psychiatry Research, Karolinska Institutet, Sweden.

Simon Cervenka, Department of Clinical Neuroscience, Center for Psychiatry Research, Karolinska Institutet, Sweden.

Lena Flyckt, Department of Clinical Neuroscience, Center for Psychiatry Research, Karolinska Institutet, Sweden.

Karin Collste, Department of Clinical Neuroscience, Center for Psychiatry Research, Karolinska Institutet, Sweden.

Pauliina Ikonen, Department of Clinical Neuroscience, Center for Psychiatry Research, Karolinska Institutet, Sweden.

Fredrik Piehl, Neuroimmunology Unit, Department of Clinical Neuroscience, Karolinska Institutet, Sweden.

Ingrid Agartz, NORMENT, Division of Mental Health and Addiction, KG Jebsen Centre for Psychosis Research, University of Oslo and Department of Psychiatric Research, Diakonhjemmet Hospital, Norway; Center for Psychiatric Research, Department of Clinical Neuroscience, Karolinska Institutet, Sweden.
